# Textbook of Insanity Based on Clinical Observations

**Published:** 1905-04

**Authors:** 


### BOOK REVIEWS.

TEXTBOOK OF INSANITY BASED ON CLINICAL OBSERVATIONS. For Practitioners and Students of Medicine. By Dr. R. VON KRAFFT-EBING, late Professor of Psychiatry and Nervous Diseases in the University of Vienna. Authorised Translation from the Latest German Edition by Charles Gilbert Chaddock, M.D., Professor of Diseases of the Nervous System in the Marion-Sims-Beaumont College of Medicine, Medical Department of Saint Louis University, Saint Louis. With an Introduction by Frederick Peterson, M.D., President of the New York State Commission in Lunacy. Pages xvi.-638. Royal octavo. Philadelphia: F. A. Davis Company. 1905. (Cloth, $4.00 net; half russia, $5.00 net.)

Krafft-Ebing's name has been associated with psychiatry since 1873, and in 1875 his first book on forensic psychiatry appeared. In this he taught the principle that crime was an act of will. He tried to explain inductively how the deed or criminal act was the result of a condition in the criminal which should be the task of the physician to establish. From this position, maintained for over thirty years, we find the author has not receded. In this country we are not so prone to follow the lead, that criminality is the result of disease, so much as we are the idea that it is the result of vicious training and environment.

Krafft-Ebing has for years interested himself to such a degree in his studies of "Psychopathia Sexualis" that we in this country have been too apt to associate him and his work as limited to this field. In judging of the value of this book, and of the great opportunities which the distinguished author enjoyed, and which fitted him for the preparation of his work, we must remember that he is the successor of the late Professor Meynert in the most important professorship of psychiatry in Germany.

The author first considers in his introduction the historic review of the development of psychiatry as a science, treating of psychiatry in ancient times, and in the middle ages, and finally of the rehabilitation of psychiatry at the end of the eighteenth century.

He defines mental disease from an anatomic standpoint, as a diffuse disease of the cerebral cortex, consisting of changes which may vary from mere alterations of cortical nutrition to gross changes of structure, especially inflammatory and degenerative in character. He lays down the dictum that a diffuse disease of the cerebral cortex must necessarily induce one of consciousness and the psychic personality, hence the psychosis must not be considered simply as a disease of the brain, but also as an abnormal alteration of the personality.

His study of the causes of insanity is most elaborate, occupying as it does, over sixty pages. He divides these into predisposing causes, which may be general or individual, and accessory causes, which may be psychic or physical.

Among the individual predisposing causes it is interesting to note that he speaks of the military life. This we have had reported from the Philippines as being a cause of insanity among our soldiers in the field. On this point he says homesickness, physical exertion and brutal treatment are effective, insults that must be endured, owing to discipline. Of insanity among prisoners he makes a stronger statement than is usually made of prisoners of the United States. He says the great frequency of insanity among prisoners is a statistic fact. The causes of this lie not exclusively from the imprisonment, but essentially in the former manner of life and certain predispositions that affect criminals. In this country from such statistics as we have had access to we believe our criminals are separated from the insane before trial in a very large majority of cases, and that with us great frequency of insanity among prisoners is not a statistic fact.

He agrees with all authors that heredity is by far the most important cause of insanity. He calls attention to the important rôle of acute diseases as a cause of insanity. He says acute diseases in which a high temperature is quickly reached with a sudden critical fall, are not unimportant causes of disturbance of the mental function. In such cases elementary disturbances of the mind are common.

When insanity is due to intoxication, alcohol takes the first rank. In his statistics the number of cases of insanity due to drink varies between 1-9 and 1-3 of all admissions to asylums. Of other poisons besides alcohol, he enumerates opium, cannabis indica, and atropine. Of this last drug he quotes Kowalewsky, who affirms there was an atropine psychosis in a patient into whose eyes atropine had been dropped. In Buffalo this hallucinatory insanity after the use of a mydriatic has been observed and recorded.

He reports cases of insanity after the use of salicylic acid, iodoform, ergot, tobacco, metallic poisons, and insanity from the inhaling of poisonous gases.

His section or classification of the forms of insanity is most valuable to the student. It is abundantly illustrated with cases, eighty-one such being given in extenso, to illustrate the various forms of insanity. The descriptions are graphic and include the methods of treatment, nor is the outlook for the insane apparently any better now than it has been for years. So far as statistics in insanity are concerned, the proportion of cures is still woefully small.

It is interesting to note in view of his prolonged studies of hypnotism, that now he devotes less than one page to the treatment of insanity by hypnotic suggestion. Of this method he now says for those experienced in psychiatry, hypnotism can only be employed with doubt as to the result, but that symptomatic manifestations of abnormal instinctive impulses, especially in those that are sexual or alcoholic, or with the desire for morphine and cocaine, are influenced by suggestive treatment. It is worthy of note that the author does not say how much they are influenced by suggestive treatment, nor does he give any statistics which will inform us of the subject.

The American psychitrists will be glad of an opportunity to have this work of Krafft-Ebing, which has for so long been the clinical textbook in the universities of Europe, and although not a modern treatise, it is, nevertheless, one of the best clinical books on insanity, and places the facts of pathological psychology more clearly than many of the modern writers. We have no hesitation, then, in recommending this excellent translation to those interested in this subject. J. W. P.

